# SHP2 phosphatase as a novel therapeutic target for melanoma treatment

**DOI:** 10.18632/oncotarget.12074

**Published:** 2016-09-16

**Authors:** Ruo-Yu Zhang, Zhi-Hong Yu, Lifan Zeng, Sheng Zhang, Yunpeng Bai, Jinmin Miao, Lan Chen, Jingwu Xie, Zhong-Yin Zhang

**Affiliations:** ^1^ Department of Medicinal Chemistry and Molecular Pharmacology, Center for Cancer Research, and Institute for Drug Discovery, Purdue University, West Lafayette, IN 47907, USA; ^2^ Department of Biochemistry and Molecular Biology, Indiana University School of Medicine, Indianapolis, IN 46202, USA; ^3^ Department of Pediatrics, Indiana University School of Medicine, Indianapolis, IN 46202, USA; ^4^ Department of Chemistry, Center for Cancer Research, and Institute for Drug Discovery, Purdue University, West Lafayette, IN 47907, USA

**Keywords:** melanoma, protein tyrosine phosphatase, SHP2, SHP2 inhibitor, drug discovery

## Abstract

Melanoma ranks among the most aggressive and deadly human cancers. Although a number of targeted therapies are available, they are effective only in a subset of patients and the emergence of drug resistance often reduces durable responses. Thus there is an urgent need to identify new therapeutic targets and develop more potent pharmacological agents for melanoma treatment. Herein we report that SHP2 levels are frequently elevated in melanoma, and high SHP2 expression is significantly associated with more metastatic phenotype and poorer prognosis. We show that SHP2 promotes melanoma cell viability, motility, and anchorage-independent growth, through activation of both ERK1/2 and AKT signaling pathways. We demonstrate that SHP2 inhibitor 11a-1 effectively blocks SHP2-mediated ERK1/2 and AKT activation and attenuates melanoma cell viability, migration and colony formation. Most importantly, SHP2 inhibitor 11a-1 suppresses xenografted melanoma tumor growth, as a result of reduced tumor cell proliferation and enhanced tumor cell apoptosis. Taken together, our data reveal SHP2 as a novel target for melanoma and suggest SHP2 inhibitors as potential novel therapeutic agents for melanoma treatment.

## INTRODUCTION

Melanoma is a malignant tumor of melanocytes and ranks among the most common cancers in the United States. Melanoma is also among the most aggressive human cancers, with 1- and 2-year survival rates in patients with metastatic melanoma of ~25% and 10%, respectively, and a median overall survival of 6 months. New treatments for melanoma have been developed over the past few years, including a number of molecular targeted and immunotherapeutic approaches. However, these targeted approaches are usually effective only in a subset of patients and the emergence of drug resistance further reduces durable responses [1]. These limitations highlight the need for better understanding of the mechanisms of pathogenesis and acquired drug resistance in melanoma, identification of new therapeutic target, and discovery of more potent pharmacological agents.

Several key signaling pathways and molecules have been implicated in the onset and progression of melanoma [1–3]. The extracellular signal-regulated kinase (ERK1/2) pathway, which controls key cellular processes such as proliferation, invasion, and survival, is frequently activated in human cancers including melanoma. Mutations of components in this pathway leading to constitutive activation of ERK1/2 are often associated with melanoma development [4]. The PI3K-AKT pathway is also implicated in melanoma. Constitutive activation of PI3K-AKT pathway facilitates melanoma progression, possibly by enhanced cell survival [5]. Phospho-AKT level increases dramatically during melanoma progression and invasion and is inversely correlated with patient survival [6]. Several growth factor receptors acting upstream of ERK1/2 and PI3K-AKT cascades are also implicated in melanoma. For example, hepatocyte growth factor receptor, c-Met, is expressed and activated in melanoma tissues and cell lines [7]. Overexpression of c-Met is associated with melanoma progression and metastasis [8, 9], and constitutive activation of c-Met signaling promotes melanoma metastasis in mice [10]. Stem cell factor receptor, c-KIT, is an important receptor tyrosine kinase involved in melanocyte development, migration, and survival. Melanoma arising from mucosal, acral, and chronically sun-damaged sites commonly has amplifications or activating mutations in KIT [11].

SHP2, encoded by *PTPN11*, is a protein tyrosine phosphatase that promotes both ERK1/2 and PI3K-AKT signaling [12–15]. Germline mutations in *PTPN11* cause Noonan and LEOPARD syndromes [16, 17], while somatic mutations in *PTPN11* have been linked to childhood and adult malignancies [18, 19]. Increased SHP2 expression has been recognized as a prognostic and predictive marker in gastric, breast, oral, prostate, lung, head and neck, thyroid, liver and pancreatic cancers [20–28]. Most recently, SHP2 overexpression and mutations were also found in melanoma patient samples [29–31]. In addition, given the obligatory requirement of SHP2 in signaling pathways mediated by receptor tyrosine kinases, many of which are up-regulated in melanoma, SHP2 may also be required for melanoma pathogenesis and progression. In the current study, we provide evidence that SHP2 promotes melanoma cell viability, motility, migration and anchorage-independent growth, likely due to the observed positive regulation of ERK1/2 and AKT pathways. We demonstrate that a specific SHP2 inhibitor 11a-1 [32] effectively suppresses SHP2's positive effects on multiple cellular processes as well as ERK1/2 and AKT signaling pathways in melanoma cell. Most importantly, 11a-1 significantly suppresses xenografted melanoma tumor growth, validating SHP2 as a novel target for melanoma and SHP2 inhibitors as potential therapeutic avenue for melanoma treatment.

## RESULTS

### The clinical relevance of SHP2 in melanoma

**Figure 1:**
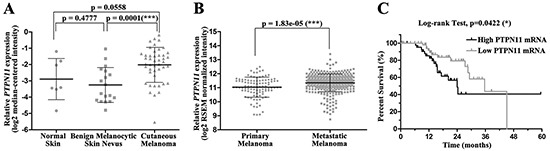
SHP2 shows clinical significance in melanoma **A.** *PTPN11* mRNA is up-regulated in melanoma tissues. Data are shown as mean±SD. **B.** *PTPN11* mRNA is further elevated in metastatic melanoma versus primary melanoma. Data are shown as mean±SD. **C.** Higher *PTPN11* mRNA shortens melanoma patient survival. *PTPN11* mRNA and survival time were extracted respectively from RSEM normalized mRNAseq and merged clinical data for patients in TCGA skin cutaneous melanoma study.

To explore the clinical significance of SHP2 in melanoma, we analyzed *PTPN11* mRNA level in the *Oncomine* database [33, 34]. *PTPN11* mRNA level in cutaneous melanoma appeared higher than that in normal skin although without statistical significance (*p* = 0.0558), probably due to the small sample size for normal skin tissue (Figure 1A). Notably, when compared to the benign melanocytic skin nevus, which is commonly viewed as the precursor of melanoma, *PTPN11* mRNA was significantly up-regulated in melanoma (Figure 1A). We also compared *PTPN11* mRNA level between primary and metastatic melanoma samples in the skin cutaneous melanoma dataset in the TCGA database [35, 36]. As shown in Figure 1B, *PTPN11* mRNA is significantly elevated in metastatic melanoma versus primary melanoma. Most importantly, the Kaplan-Meier survival analysis for patients in this dataset revealed that the high *PTPN11* mRNA expression group showed significantly shorter overall survival time compared to the low *PTPN11* mRNA group, with a median survival of 24.03 months versus 35.67 months (Figure 1C). Collectively, these clinical data suggest that SHP2 may play a role in melanoma onset and progression, and thus targeting SHP2 may be beneficial for melanoma treatment.

### SHP2 promotes melanoma cell viability, motility and anchorage-independent growth

**Figure 2:**
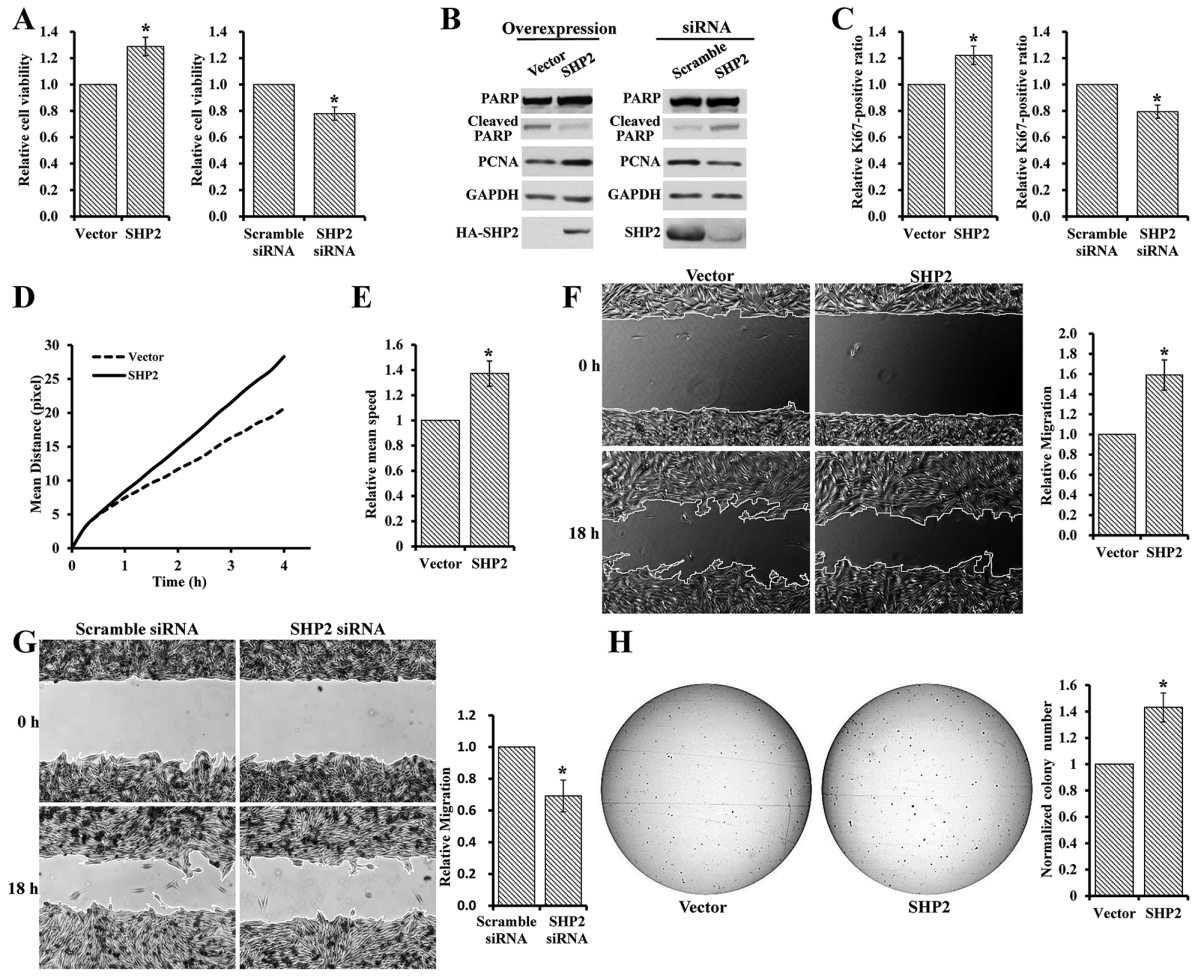
SHP2 promotes melanoma cell viability, motility, and anchorage-independent growth **A.** SHP2 overexpression enhances MeWo cell viability, and SHP2 knockdown reduces MeWo cell viability. **B.** SHP2 overexpression in MeWo cell enhances proliferation and supresses apoptosis, and SHP2 knockdown supresses proliferation and enhance apoptosis. **C.** SHP2 overexpression in MeWo cell increases Ki67-positive (i.e. proliferative) cells, and SHP2 knockdown decreases Ki67-positive cells. SHP2 overexpression promotes MeWo cell **D.** movement **E.** moving speed **F.** migration and **H.** anchorage-independent growth. **G.** SHP2 knockdown slows down the MeWo cell migration.

Although SHP2 expression is elevated in melanoma, the role of SHP2 in melanoma is unknown. To investigate the biological function of SHP2 in melanoma, we firstly examined the effect of SHP2 modulation on melanoma cell viability. To this end, we either overexpressed or knocked down SHP2 in MeWo melanoma cells and measured the cell viability by the MTT assay. As shown in Figure 2A, SHP2 overexpression increased cell viability by ~30% and SHP2 knockdown decreased that by ~20%. To gain insights into the positive role of SHP2 in melanoma cell viability, we examined the proliferation marker PCNA and apoptosis marker cleaved PARP after SHP2 overexpression or knockdown in MeWo cells (Figure 2B). PCNA was increased and cleaved PARP was reduced in SHP2 overexpressed cells. Conversely, PCNA was decreased and cleaved PARP was increased in SHP2 knockdown cells. Together, the data indicates that SHP2 promotes melanoma cell viability through enhancing cell proliferation and suppressing apoptosis. To strengthen this conclusion, SHP2 overexpression or knockdown MeWo cells were stained with Hoechst and anti-Ki67 antibody, and the Ki67-positive cells were visualized and analyzed by an ArrayScan high-content screening system. Consistently, the number of Ki67-positive cells increased following SHP2 overexpression and decreased after SHP2 knockdown (Figure 2C).

Increased cell motility is one of the defining characteristics of invasive tumors and is crucial to tumor invasion and metastasis [37]. To assess the effect of SHP2 on melanoma cell motility, MeWo cells overexpressing SHP2 or vector control were stained with Hoechst and monitored by an ArrayScan high-content screening system. Compared to vector control, SHP2 overexpression clearly increased the mean moving distance of melanoma cells at the same time point (Figure 2D) and led to ~1.4-fold increase in the mean speed of cell movement by the end of 4 hours observation window (Figure 2E). We also carried out wound healing experiment to further evaluate the effect of SHP2 on cell motility. As shown in Figure 2F and 2G, SHP2 overexpression sped up MeWo cell migration by 1.6 folds, whereas SHP2 knockdown slowed down MeWo cell migration by 30%.

Anchorage-independent growth is a hallmark of oncogenic transformation and a key characteristic of cancer cells [38], and the *in vitro* anchorage-independent growth of cancer cells has been connected with tumor cell aggressiveness *in vivo* such as tumorigenic and metastatic potentials [39]. Thus we also investigated the effect of SHP2 on anchorage-independent growth of MeWo cell using colony formation assay. Compared to vector control, larger and 45% more colonies were observed in SHP2 overexpressing melanoma cells (Figure 2H). Collectively, the above results indicate that SHP2 promotes melanoma cell viability, motility, and anchorage-independent growth.

### SHP2 positively regulates ERK1/2 and PI3K/AKT pathway in melanoma cells

**Figure 3:**
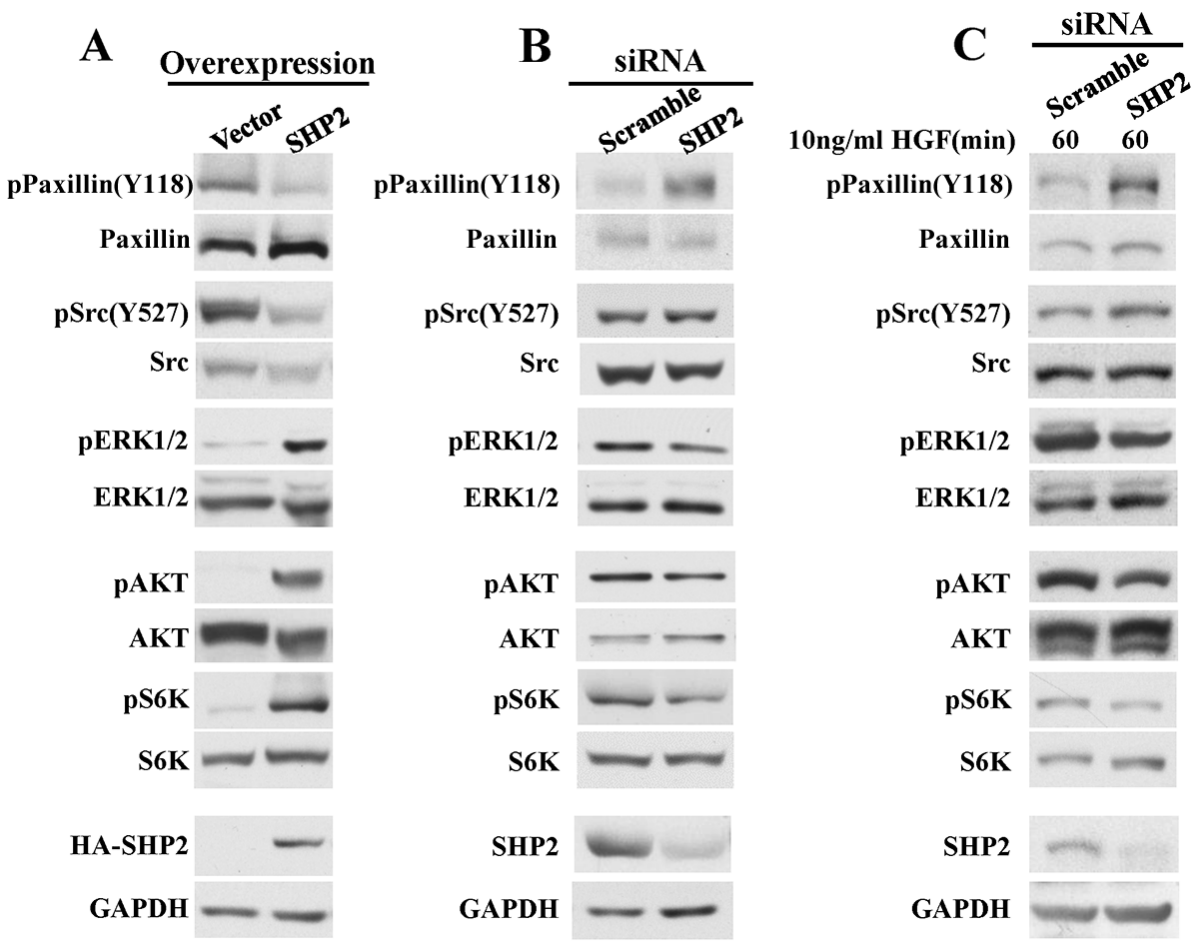
SHP2 positively regulate ERK1/2 and AKT signaling pathways **A.** SHP2 overexpression promotes dephosphorylation of pPaxillin(Y118) and pSrc(Y527), as well as ERK1/2, AKT and S6K activation. Consistently, SHP2 knockdown attenuates dephosphorylation of pPaxillin(Y118) and pSrc(Y527), as well as ERK1/2, AKT and S6K activation under **B.** non-stimulation or **C.** HGF stimulation.

ERK1/2 and PI3K/AKT are the two major signal pathways implicated in melanoma pathogenesis and progression [2], and SHP2 is a recognized positive regulator of Ras, which activates both pathways [12–15]. Thus, we examined the effect of SHP2 on both ERK1/2 and PI3K-AKT pathways in MeWo cells. As shown in Figure 3A, compared to the vector control, ERK1/2 activation is significantly enhanced in SHP2 overexpression cells. We then assessed the phosphorylation of Paxllin (Y118), a physiological substrate of SHP2 required for Src activation [40]. SHP2 promotes the activation of Src family kinases through dephosphorylation of Paxillin (Y118), thereby preventing Csk from phosphorylating the C-terminal inhibitory Tyr527 in Src kinase [40, 41]. As expected, the pPaxillin (Y118) and pSrc (Y527) levels are decreased in SHP2 overexpressed cells, thereby contributing to ERK1/2 activation. SHP2 overexpression also promotes AKT activation, leading to enhanced phosphorylation of the downstream S6K which might contribute to the positive role of SHP2 on cell proliferation. To consolidate the above effects of SHP2 on both ERK1/2 and AKT signal pathways, we also determined the effects of SHP2 knockdown in MeWo cells. As expected, both pPaxillin (Y118) and pSrc (Y527) are elevated, and ERK1/2, AKT and S6K activation are diminished when SHP2 level is reduced (Figure 3B). Since c-Met is a receptor tyrosine kinase involved in melanoma [7, 8, 10], we further evaluated whether SHP2 could affect aforementioned signals upon HGF stimulation (Figure 3C). As expected, SHP2 knockdown diminishes the dephosphorylation of Paxillin pY118 and Src pY527, and attenuates ERK1/2, AKT and S6K activation after HGF stimulation, and the alterations on these signaling molecules are more obvious compared to those without HGF stimulation in Figure 3B. Collectively, these results reveal that SHP2 plays a positive role in promoting ERK1/2 and AKT pathways in MeWo melanoma cells.

### SHP2 inhibitor 11a-1 reduces melanoma cell viability, motility, and anchorage-independent growth

**Figure 4:**
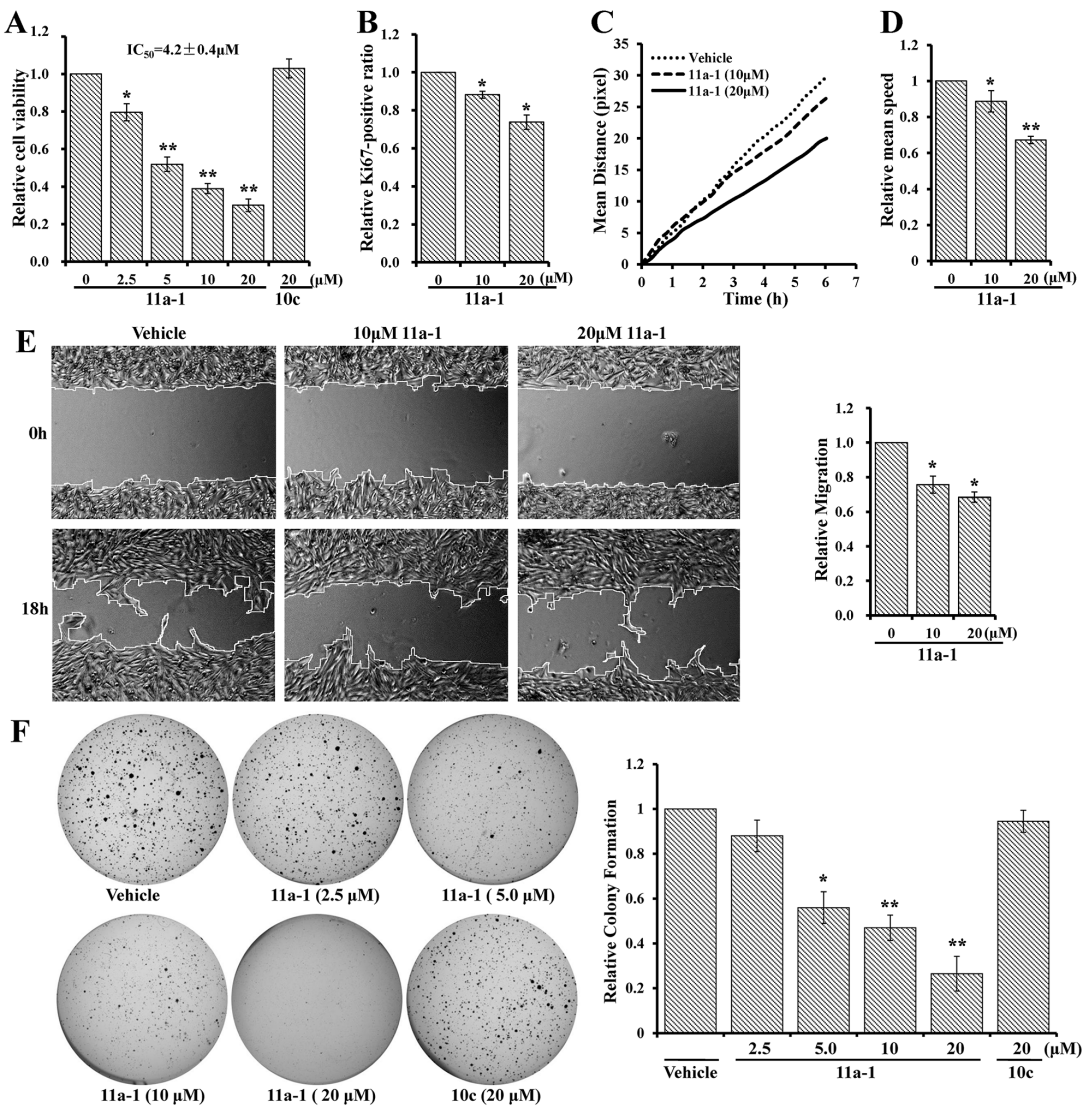
SHP2 inhibitor 11a-1 effectively inhibit mutiple processes in melanoma cell 11a-1 dose-dependently inhibits MeWo cell **A.** viability **B.** proliferation **C&D.** motility **E.** migration and **F.** anchorage-independent growth.

Given the observed positive role of SHP2 in promoting melanoma cell viability, motility, anchorage-independent growth, as well as ERK1/2 and PI3K/AKT pathway activation, we speculated that pharmacological inhibition of SHP2 could counteract these positive effects thereby benefit melanoma treatment. Thus we used a potent and specific SHP2 inhibitor 11a-1 [32] to validate the potential of targeting SHP2 for melanoma treatment. We first evaluated 11a-1's cellular effect on melanoma cell viability by the MTT assay. As shown in Figure 4A, 11a-1 dose-dependently inhibits MeWo cell viability with an EC_50_ of 4.2 μM and a maximum inhibition of ~70%, but a structurally-related, inactive analog 10c [32] did not show any inhibition up to 20 μM, indicating that 11a-1 suppress melanoma cell viability through inhibiting SHP2. Similar inhibitory effect for 11a-1 on cell viability was also observed in a mouse melanoma cell line B16F10, with an EC_50_ of 5.1 μM and a maximum inhibition of ~95% (Supplementary Figure S1A). To further ascertain target engagement by 11a-1, we also determined the effect of 11a-1 on NIH 3T3 cell line which is non-tumorigenic and with significantly lower SHP2 expression in comparison to that in MeWo cell (Supplementary Figure S2A). As shown in Supplementary Figure S2B, the EC_50_ value of 11a-1 on NIH 3T3 cell viability (49.8 ± 5.2 μM) is more than 10 fold higher than that on MeWo cells, indicating that 11a-1 shows less toxicity to normal cells. To further consolidate the target engagement of SHP2, we evaluated two additional SHP2 inhibitors, one is a structurally-related active site-directed SHP2 inhibitor IIB08 (IC_50_ = 5.5 μM) [42], and the other is a structurally-unrelated SHP2 inhibitor SHP099 (IC_50_ = 0.07 μM) with a different mode of action, namely allosteric inhibition, reported most recently by Novartis [43]. As shown in Supplementary Figure S2C and S2D, IIB08 and SHP099 dose-dependently inhibit MeWo cell viability and SHP2 mediated ERK1/2 activation. Collectively, these results indicate that 11a-1 inhibits MeWo cell viability through specifically inhibiting SHP2 mediated signaling pathways.

We further examined whether 11a-1 can neutralize SHP2's positive effect on other cellular processes. MeWo cells treated with vehicle or 11a-1 were stained by Hoechst and anti-Ki67 antibody, visualized by an ArrayScan high-content screening system and the Ki67-positive ratio was calculated to quantify cell proliferation. 11a-1 decreases the Ki67-positive ratio by 12% and 26% respectively at 10 and 20 μM, showing a dose-dependent inhibition on cell proliferation (Figure 4B). To assess the effect of SHP2 inhibition on cell motility, we treated MeWo cells with vehicle or 11a-1, stained cell nucleus with Hoechst and monitored the mean moving distance of cells by an ArrayScan high-content screening system. As shown in Figure 4C, 11a-1 treatment dose-dependently shortens the mean moving distance of MeWo cells. The mean speed by the end of the experiments (6 hours duration) was calculated and normalized to vehicle treatment, and 11a-1 was found to reduce the mean speed by 12% and 23% respectively at 10 and 20 μM (Figure 4D). Similar inhibitory effects were also observed in B16F10 cells (Supplementary Figure S1B & S1C). We then proceeded to evaluate the effect of 11a-1 on cell migration by wound healing assay. As show in Figure 4E, 11a-1 slows down MeWo cell migration by ~25% and ~33% respectively at 10 and 20 μM. Finally, we examined the effects of 11a-1 on anchorage-independent growth of melanoma cells. As expected, 11a-1 effectively inhibits MeWo cell anchorage-independent growth in a dose dependent manner, and almost completely blocks colony formation at 20 μM, while the negative control compound 10c barely shows any inhibition even at 20 μM (Figure 4F). This same inhibitory effect on anchorage-independent growth by 11a-1 was also observed with the mouse melanoma cell B16F10 (Supplementary Figure S1D). Collectively, the results indicated that pharmacological inhibition of SHP2 by 11a-1 can effectively inhibit melanoma cell viability, motility, and anchorage-independent growth, confirming the therapeutic potential of targeting SHP2 for melanoma treatment at cellular level.

### 11a-1 attenuates SHP2-mediated signaling pathways in melanoma cell

**Figure 5:**
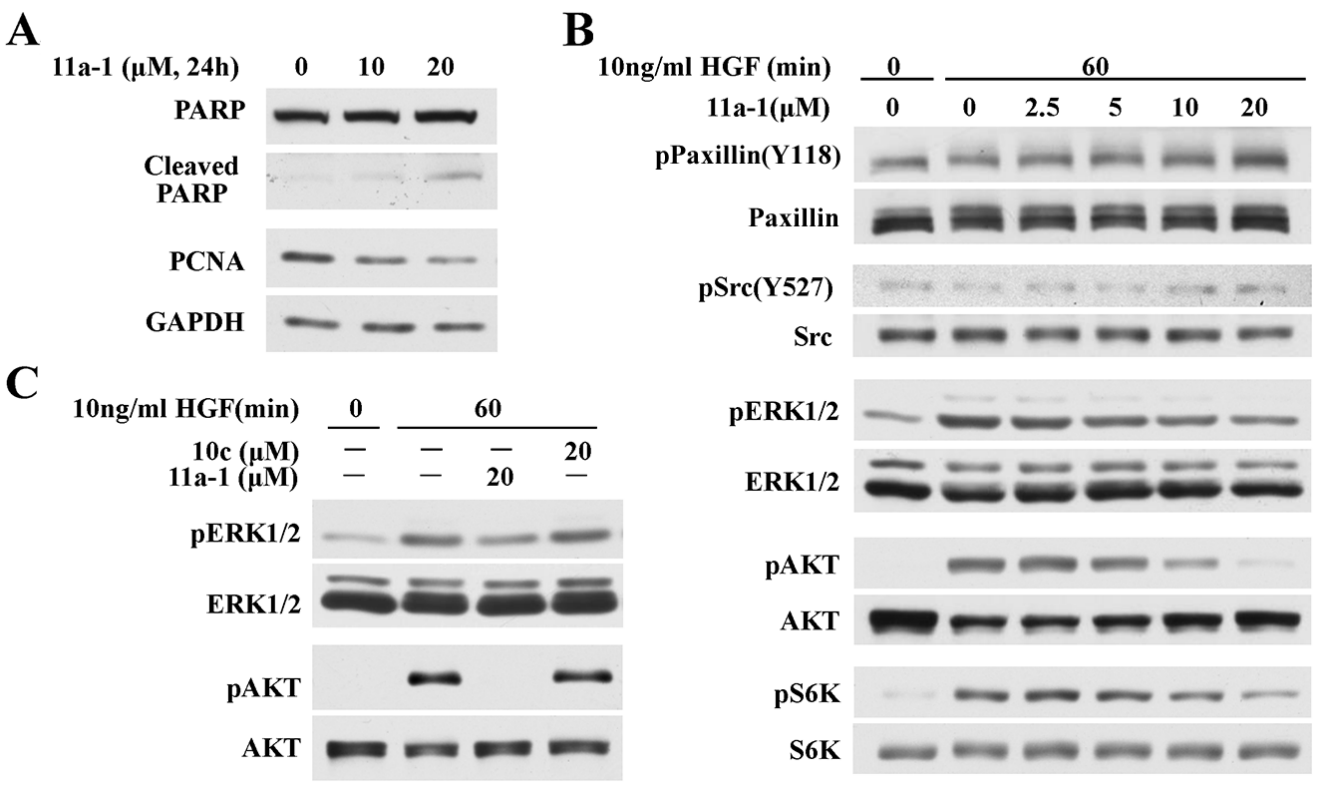
11a-1 inhibits SHP2-mediated signaling pathway **A.** 11a-1 treatment leads to decreased proliferation marker PCNA and increased apoptosis marker cleaved PARP in MeWo cell. **B.** 11a-1 inhibits SHP2-mediated ERK1/2 and AKT signaling pathway in MeWo cell. **C.** Negative control 10c has no effect on ERK1/2 and AKT activation up to 20 μM.

To delineate the molecular mechanisms underlying the mode of action of 11a-1 in melanoma cells, we performed Western blot analyses to evaluate its effects on SHP2 relevant signaling pathways. The data shows that 11a-1 decreases PCNA and increases cleaved PARP level 2 and 5.5 fold after cells were treated with 10 and 20 M 11a-1 for one day (Figure 5A), consistent with 11a-1's ability to suppress cell proliferation and induce apoptosis. Moreover, we also ascertained the effect of 11a-1 on SHP2-mediated ERK1/2 and PI3K/AKT pathway activation in MeWo cell. As shown in Figure 5B, 11a-1 dose-dependently augments the phosphorylation of Paxillin(Y118) and Src(Y527) and inhibits HGF-induced ERK1/2 activation. 11a-1 also inhibits AKT activation and consequently causes inhibition of downstream S6K activation. However, the inhibition of ERK1/2 and AKT activation were not observed after the negative control compound 10c treatment at 20 μM (Figure 5C), indicating target engagement of SHP2 in the signal transduction. Again, similar results were observed in B16F10 cells. 11a-1 inhibits HGF-induced ERK1/2 and AKT activation, but 10c shows no appreciable inhibition up to 20 μM (Supplementary Figure S3). Overall the data nicely showed that pharmacological inhibition of SHP2 by 11a-1 phenocopied SHP2 knockdown.

### 11a-1 suppresses melanoma xenograft tumor growth

**Figure 6:**
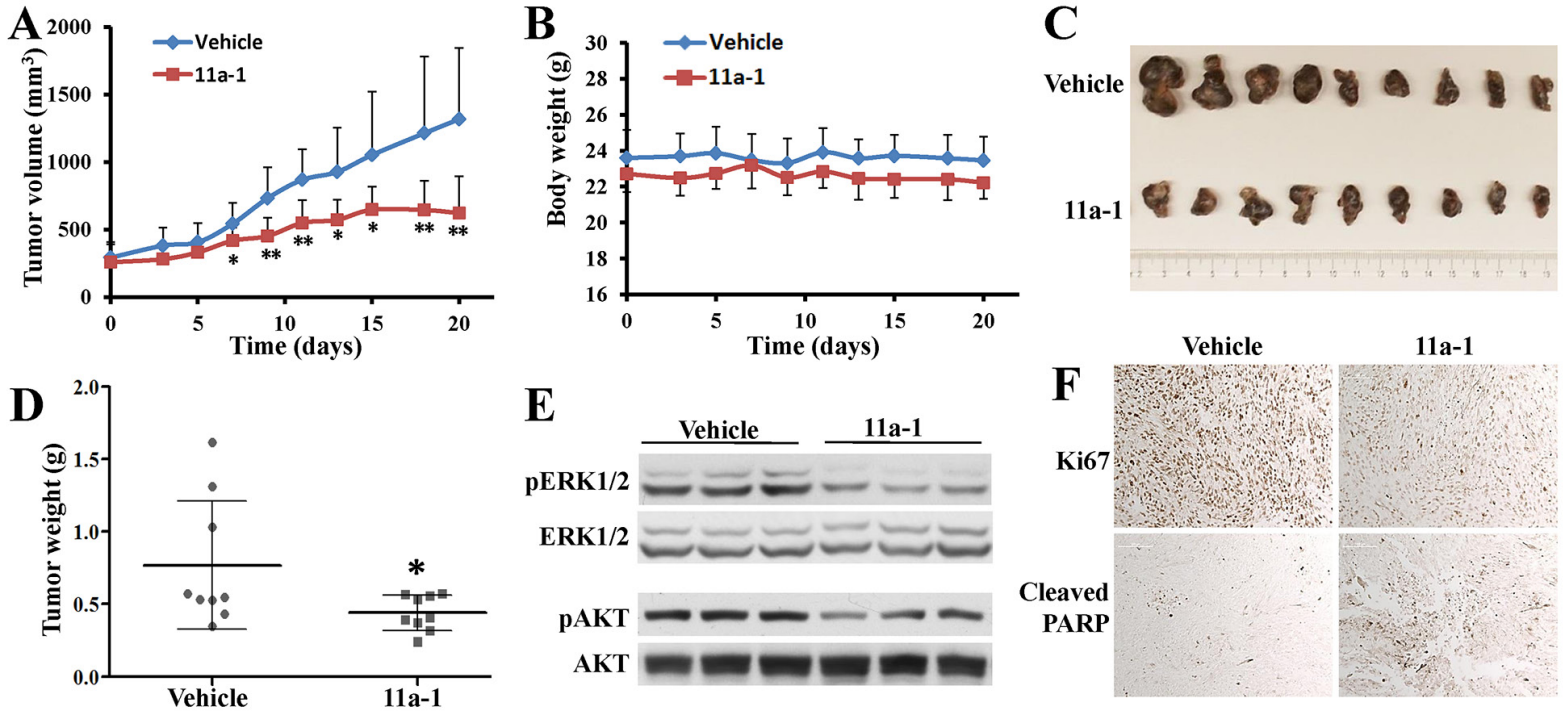
11a-1 suppresses MeWo melanoma cell xenograft tumor growth *in vivo* **A.** 11a-1 significantly suppressed tumor growth since 7 days after treatment (n=9 for each group; *: p<0.05; **: p <0.01 **B.** 11a-1 treatment didn't significantly change the body weight (n=9 for each group). **C.** 11a-1 treatment for 21 days significantly reduced tumor size. **D.** 11a-1 treatment for 21 days significantly reduced tumor weight (n=9 for each group; *: p<0.05). **E.** 11a-1 treatment led to decreased ERK1/2 and AKT activation in MeWo xenograft tumor samples. **F.** 11a-1 treatment inhibited cell proliferation and enhanced apoptosis in MeWo xenograft tumor samples.

Given the observed promising activity of 11a-1 in blocking SHP2-mediated signaling in cell-based assays, we wanted to provide further proof-of-concept for targeting SHP2 for melanoma treatment. Pharmacokinetic (PK) analysis of plasma samples from a cohort of three mice collected at 1, 3, 6, 9, and 24 hr post-intraperitoneal (IP) injection of 11a-1 showed that 11a-1 displayed a very respectable PK profile with a maximum plasma compound exposure C_max_= 0.6 μM at 1 hr and a half-life t_1/2_=9.5 hr at a 10 mg/kg single dose (Supplementary Figure S4). Given the 11a-1's excellent PK properties, we evaluated the efficacy of 11a-1 on melanoma tumor growth *in vivo* using a xenograft mouse model. MeWo cells were injected into one flank of each mouse, and the mice with grown tumors were randomly assigned to either a vehicle control group or an 11a-1 treatment group. Mice received IP injection of vehicle (DMSO) or 11a-1 (15 mg/kg) once daily, and the tumor growth was monitored for three weeks. As shown in Figure 6A, tumors in the vehicle-treated group progress steadily during the course of treatment, while tumor growth in 11a-1-treated group is significantly inhibited starting from the seventh day, and the average tumor volume is reduced 52% at day 21 compared to vehicle control. During the treatment period, the body weight of mice in both groups remained the same, indicating that 11a-1 was well tolerated with no obvious toxicity (Figure 6B). The mice were sacrificed after 21-day treatment, and the tumors were harvested and weighed. Tumors in the 11a-1 treatment group are significantly smaller than those in vehicle control group (Figure 6C). Correspondingly, the tumor weight in 11a-1 group is also significantly reduced (Figure 6D). To study the effects of 11a-1 on SHP2-mediated signaling pathways *in vivo*, tumor samples were lysed and analyzed by Western blot. Consistent with the observations in cultured MeWo cells, 11a-1 treatment leads to decreased pERK1/2 and pAKT level in xenograft tumor samples (Figure 6E). Moreover, the immunohistochemical analyses of tumor samples revealed that 11a-1 treatment reduced Ki67 level and elevated cleaved PARP level (Figure 6F), indicating that 11a-1 attenuated tumor cell proliferation and enhanced tumor cell apoptosis *in vivo*. We also evaluated the anti-melanoma effect of 11a-1 in syngeneic B16F10 melanoma xenograft mice, and found that 11a-1 significantly reduced melanoma xenograft tumor weight, as well as pERK1/2 and pAKT level in B16F10 melanoma xenograft tumor tissue (Supplementary Figure S5). Collectively, our results provide the first proof-of-concept for the therapeutic potential of targeting SHP2 for melanoma treatment.

## DISCUSSION

During the past decade, great progress in targeted therapies and immunotherapies was made for melanoma treatment. However, the extension of life achieved by these agents is only several months due to the rapid occurrence of drug resistance [44]. Moreover, treatment for metastatic melanoma, which is linked with a poor patient prognosis, still remains limited. Melanoma progression involves a complex series of events, including malfunction of many signaling pathways and key oncogenic molecules. Elucidating the intricate mechanisms that initiate and drive melanoma and identification of new therapeutic targets that might be crucial for clinical applications are likely to lead to more effective treatment strategies for melanoma.

SHP2 plays important roles in cell proliferation, migration, cell transformation, angiogenesis, and survival. SHP2 is a key signal-transducer acting downstream of most receptor tyrosine kinases including EGFR, FGFR, c-MET, Kit, PDGFR, and insulin receptor, and positively regulates Ras/ERK1/2, PI3K/AKT, JAK/STAT, JNK, FAK, NF-κB, and Wnt signaling pathways [12–15, 45]. Noticeably, some of the growth factor receptors and signaling pathways modulated by SHP2 are also implicated in melanoma pathogenesis, suggesting that SHP2 might play a role in melanoma onset or progression. To substantiate this speculation, we performed bioinformatics analyses and found that SHP2 mRNA level is elevated in melanoma compared to its precursor nevus. Moreover, high SHP2 expression is significantly associated with more metastatic phenotype and poorer prognosis, and the survival time of patients with high SHP2 level is substantially shorter than those with low SHP2 level. The correlation between SHP2 expression and clinicopathological characteristics implicates a potential role of SHP2 in melanoma. In addition, given the obligatory requirement of SHP2 in receptor tyrosine kinase pathways, inhibition of SHP2 is expected to have therapeutic applications in a wide range of cancers including melanoma.

To disclose the role of SHP2 in melanoma pathogenesis and explore the potential of targeting SHP2 for melanoma treatment, we first assessed the biological function of SHP2 in melanoma by overexpressing or knocking down SHP2 in MeWo cells. We found that SHP2 is a positive regulator of both ERK1/2 and AKT signaling, and promotes melanoma cell viability, anchorage-independent growth, and migration which are key characteristics in cancer cells progression and metastasis. These observations suggest that SHP2 acts as an oncogene involved in melanoma progression and metastasis, and support the notion that SHP2 may serve as a novel and promising target for melanoma treatment. Thereby, we proceeded to evaluate the effects of a small molecule SHP2 inhibitor 11a-1 on melanoma in cell culture and *in vivo*. Treatment of MeWo and B16F10 metastatic melanoma cell lines with 11a-1 effectively blocks SHP2-mediated ERK1/2 and AKT activation and attenuates cell viability, anchorage-independent growth, and migration. Most importantly, 11a-1 significantly suppresses melanoma xenograft tumor growth, at least through reducing tumor cell proliferation and enhancing apoptosis. Our results validate SHP2 as a novel target for melanoma and suggest pharmacological inhibition of SHP2 could be highly effective therapeutic approach for melanoma treatment.

Finally, SHP2 plays important roles in several cellular processes, and there is a large body of literature implicating SHP2 as a bona fide oncoprotein. Moreover, since SHP2 is directly downstream of receptor tyrosine kinases, targeting the growth factor pathways at the level of SHP2 might offer unique advantages in receptor tyrosine kinase inhibitor resistance settings. This is of particular interest since several of the currently proposed drug resistance mechanisms against melanoma, through upregulation of C-Raf [46], B-Raf [47], hepatocyte growth factor [48] or PDGFR [49], reactivate the signaling pathways which are mediated by SHP2. Indeed, a recent study indicates that SHP2 is a central node in intrinsic and acquired resistance to tyrosine kinase targeted cancer drugs [50]. However whether pharmacologic inhibition of SHP2 represents an effective approach for cancer therapy remains an outstanding question. Results from this study together with early reports [32, 51, 52] provide strong evidence that specific inhibitors of SHP2 can be effective anti-tumor agents. Future studies will investigate whether SHP2 inhibitors alone or in combination with kinase inhibitors can prevent or prolong the development of cancer drug resistance.

## MATERIALS AND METHODS

### Cells lines, antibodies and reagents

MeWo and B16F10 cell lines were purchased from the American Tissue Culture Collection. The following antibodies were from Cell Signaling Technology: phospho AKT (CAT. #: 4060), AKT (CAT. #: 2920), phospho ERK1/2 (CAT. #: 9101), ERK1/2 (CAT. #: 4696), phospho Paxillin (CAT. # 2541), phospho S6K (CAT. #: 9205), S6K (CAT. #: 9202), PCNA (CAT. #:2586), PARP (CAT. # 9542), cleaved PARP (CAT. # 9541), phosphor Src (CAT. #: 2105), Src (CAT. #: 2108), horseradish peroxidase (HRP)-conjugated secondary antibodies (anti-mouse, CAT. #: 7076, anti-rabbit, CAT. #: 7074). The following antibodies were from Santa Cruz Biotechnology: GAPDH (CAT. #: sc-59541), SHP2 (CAT. #: sc-280), HA (CAT. #: sc-7392). Paxillin antibody (CAT. #: 610569) was from BD Transduction Laboratories. Ki67 antibody was from Thermo Fisher Scientific (CAT. # RM-9106-S0). HGF, MTT (3-(4,5-Dimethylthiazol- 2-yl)-2,5-diphenyltetrazolium bromide) were obtained from Sigma and prepared according to the manufacture's instruction. Eagle's Minimum Essential Medium (EMEM) and Dulbecco's Modified Eagle's Medium (DMEM) were from Corning, fetal bovine serum was from HyClone. SuperSignal West Dura Luminol/Enhancer solution was from Thermo Fisher Scientific. Lipofectamine® RNAiMAX and Lipofectamine® 2000 Transfection reagent was from Invitrogen Life Technologies.

### Cell culture

MeWo cells were cultured at 37°C and 5% CO2 in EMEM supplemented with 10% fetal bovine serum. B16F10 cells were cultured at 37°C and 5% CO2 in DMEM supplemented with 10% fetal bovine serum. To generate stably-transfected SHP2 cell lines, MeWo cells were transfected with pcDNA3.1-HA-SHP2 using Lipofectamine® 2000 transfection reagent according to manufacturer's instruction, the transfected cells confer resistance to G418 thereby were selected in growth medium containing 1 mg/ml G418. Single-cell colonies were selected, expanded, and used in some experiments.

### Immunoblot analysis

The cell lysates were resolved by SDS-PAGE and transferred onto nitrocellulose membranes. Membranes were blotted with various antibodies, and the blots were developed by the enhanced chemiluminescence technique using the SuperSignal West Pico Chemiluminescent substrate. Data shown in Figures is a representative result from one of multiple repeated experiments.

### Cell viability assay

~4×10^3^ cells were seeded in each well of 96-well plates. After treatment with different concentration of 11a-1 for 2 days in 5% FBS containing medium, or incubation of overexpressed or knock down cells for 3 days in 10% FBS containing medium, cells were incubated with 50 g/ml MTT for 3~4 hours. Then the culture medium was removed, DMSO was added to dissolve the formazan crystals. Wells containing only media were used for background correction. The optical density was measured spectrophotometrically at 540 nm.

### Cell motility assay

~6×10^3^cells /well were seeded in each well of a 96-well plate. On the second day, 1μg/mL of Hoechst 33342 (Thermo Scientific) was used to label the nuclei for 15min, and Thermo Scientific ArrayScan XTI Live High Content Platform was then used for live-cell tracking, image data were collected every 15 min and the motility of the cells was assessed over 4~6 h.

### RNA interference studies

Small interfering RNA (siRNA) specific for SHP2 (5′- PCACGCAUGACGCCAUAUUCTT-3′) and scrambled SHP2 siRNA (5′- PGCACGACCGCC UUAUAACUTT-3′) were synthesized by Dharmacon Research. siRNAs were transfected into MeWo cells using Lipofectamine® RNAiMAX Reagent according to manufacturer's instruction.

### Immunofluorescence analysis

Cells were seeded in 96-well plates, fixed with 4% paraformaldehyde in phosphate-buffered saline (PBS) for 10 min at room temperature, permeabilized with 0.1% Triton X-100 in PBS for 90 seconds, and blocked with 0.1% BSA in PBS for 1 hour. 0.1% BSA in PBS containing Ki67 antibody was applied overnight at 4°C, followed by 1h incubation with anti-rabbit Alexa 488 immunoglobulin G (Jackson ImmunoResearch Laboratories). Then DNA staining with 5μg/mL Hoechst (Sigma) for another 15min was used for identification of cell nuclei. After washing with PBS, each well was filled with PBS. The fraction of Ki67 possitive cells were quantified by high content immunofluorescence microscope (Arrayscan™ XTI Infinity High Content Platform). In immunofluorescence analysis assessing the effect of 11a-1 on cell proliferation, cells were treated with 11a-1 or DMSO for 1 day before being fixed with 4% paraformaldehyde contained PBS.

### Wound healing assay

The uniform scratches were created using silicon culture inserts (Ibidi, Germany) with two individual wells for cell seeding according to the manufacturer's instruction. After removing the inserts, cells were washed to remove the floating cells, and fresh medium containing 5% FBS was added. The photos were captured by EVOS FL cell imaging system (Life Technologies) at the indicated time points. The wound area was measured using MRI wound healing tool in Image J software, and the relative migration was calculated as described by Xia et al [53]: measure the initial wound area at 0 h as A_0_ and the area at time point i as A_i_, then the cell migration area at time point i is defined as A_migration_(i) = A_i_ − A_0_, and the relative migration shown in Figures was calculated by normalizing the A_migration_(i) for SHP2 versus vector overexpression cells (Figure 2F), or SHP2 siRNA versus scramble siRNA transfected cells (Figure 2G). In wound healing assay assessing the effect of 11a-1 on migration, 11a-1 or DMSO were added to fresh medium containing 5% FBS.

### Soft agar colony formation assay

Cells were suspended in 0.3% agar containing medium and plated on a layer of 0.6% agar in medium in 12-well culture plates. After incubation for 4 weeks (Figure 2H) or 6 weeks (Figure 4F), colonies were stained with crystal violet and scanned with a scanner, the numbers of colonies and the percentage of colony-covered area were counted using Image J software.

### Animal dosing and sample collection for pharmacokinetic studies

Three mice were administered a single *IP* dose of 11a-1 at 10 mg/kg. At different time points (1 hour, 3 hours, 6 hours, 9 hours and 24 hours), blood samples (50 μl) from each mouse were collected and mixed with EDTA (50 mM, 50 μl). The samples were then mixed with 200 μl acetonitrile and centrifuged at 21,100 g for 5 minutes. The supernatants were collected and subjected to liquid chromatography/mass spectrometry analysis.

### Liquid chromatography/mass spectrometry analysis

The Liquid Chromatography/Mass Spectrometry (LC/MS) analysis was carried out on a Agilent 1200 analytic HPLC system and an Agilent 6130 Quadrupole MS detector, equipped with a Phenomenex Kinetex 2.6u XB-C18 column (2.5 μm, 4.6 mm X 50 mm), eluted with 0-100% MeOH-H2O with 0.1% (w/v) ammonium bicarbonate at 0.8 mL/min flow-rate (gradient method: 9.0 min 0-90% MeOH linear gradient, followed by 3 min 90-100% MeOH, followed by 3 min 100% MeOH), MS detector were set at single ion mode (SIM), monitoring the negative charge 636 (M-1). The detection limit for 11a-1 is 50 nM at 2 μl sample injection.

### Tumor xenograft experiments

NSG (NOD/scid/IL2R^gnull^) mice were purchased from In Vivo Therapeutics Core of the Indiana University Simon Cancer Center. Xenograft experiments were performed in compliance with the relevant laws and guidelines set forth by the Institutional Laboratory Animal Care and Use Committee of Indiana University. All mice were housed under pathogen-free conditions in the animal facility and received autoclaved water and food. 10-12 weeks old NSG mice were used in the study. MeWo cells were suspended in phosphate-buffered saline (PBS) at 8×10^7^ cells/ml. A total of 8×10^6^ cells (100 μl) were subcutaneously implanted into both left and right flank using a 27-gauge needle. Tumor size was measured with calipers. Tumor volume (V) was determined by the equation V = (L × W^2^) × 0.5, in which L is the length and W is the width of the tumor. When tumors reached volumes of ~200 mm^3^, the mice showing tumors were randomly assigned to a control group and an 11a-1 group, and daily intraperitoneal injection of either vehicle or 15 mg/kg 11a-1 was performed, and the tumor growth was monitored for 3 weeks. Mice were sacrificed after injection for 21 days, and tumors were removed, weighted, as well as subjected to immunohistological and biochemical analysis.

### Immunohistochemistry analysis

Harvested tissues were fixed in 4% paraformaldehyde overnight at 4°C, embedded in paraffin, serially sectioned (7 μm), de-paraffined sections were incubated with diluted antibodies at 4°C overnight. Signals were detected by VECTASTAIN Elite ABC kit and developed using DAB substrate from Vector laboratory (Burlingame, CA). All the antibodies used were from Cell Signaling Technology. Images were captured on a Leica DM2500 stereomicroscope.

### Statistical analysis

In clinical relevance study of SHP2 in melanoma, the data was downloaded from *Oncomine* or *TCGA* database. The Student's t-test was used to assess the significance of differences between groups. Survival analysis was performed according to the Kaplan-Meier method and the log-rank test was used to assess the significance. For all cell-based assays, each experiment was repeated for 2~3 times, the representative result was shown in figures, the bar chart represented mean value ± standard deviation, and the Student's t-test was used to measure the significance. In xenograft tumors experiments, tumor volumes at different time point, as well as final tumor weights were compared between 11a-1 and vehicle treated group, the statistical significance was evaluated using the Student's t-test. In all statistical analysis, p-value less than 0.05 was considered statistically significant, and the statistical significance were represented in figures as * (p < 0.05), ** (p < 0.01) or *** (p < 0.005).

## SUPPLEMENTARY MATERIALS FIGURES


