# Supplementary material for: SHP2 phosphatase as a novel therapeutic target for melanoma treatment
Source: Oncotarget. 2016 Sep 16;7(45):73817–29. doi: 10.18632/oncotarget.12074 (PMC5342016; doi:10.18632/oncotarget.12074)
Supplement: Supplementary file 1 [file oncotarget-07-73817-s001.pdf]

SHP2 phosphatase as a novel therapeutic target for melanoma treatment

SUPPLEMENTARY FIGURES

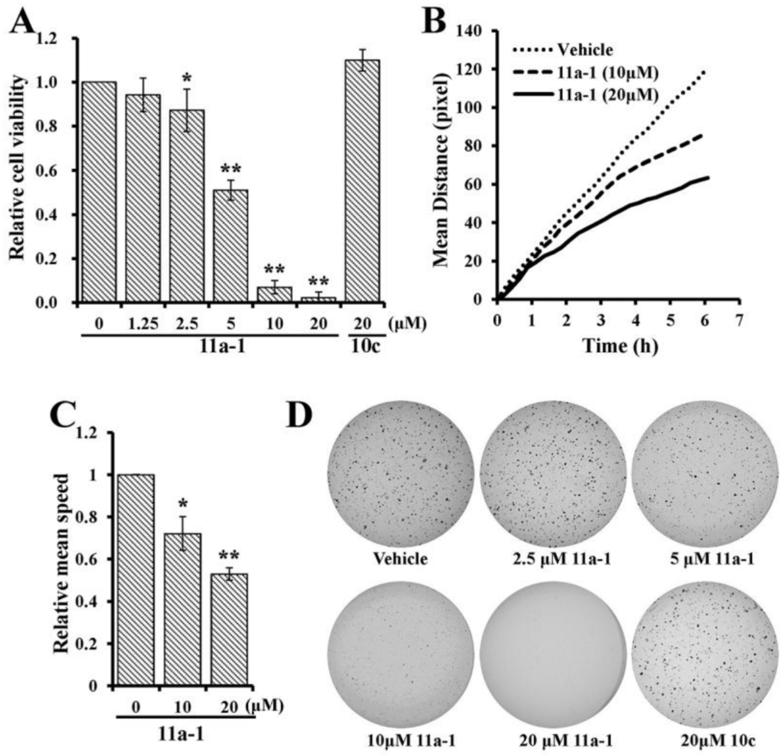

Supplementary Figure S1: SHP2 inhibitor 11a-1 inhibits A. cell viability **B.** mean moving distance **C.** mean moving speed and **D.** anchorage-independent growth in mouse melanoma B16F10 cells.

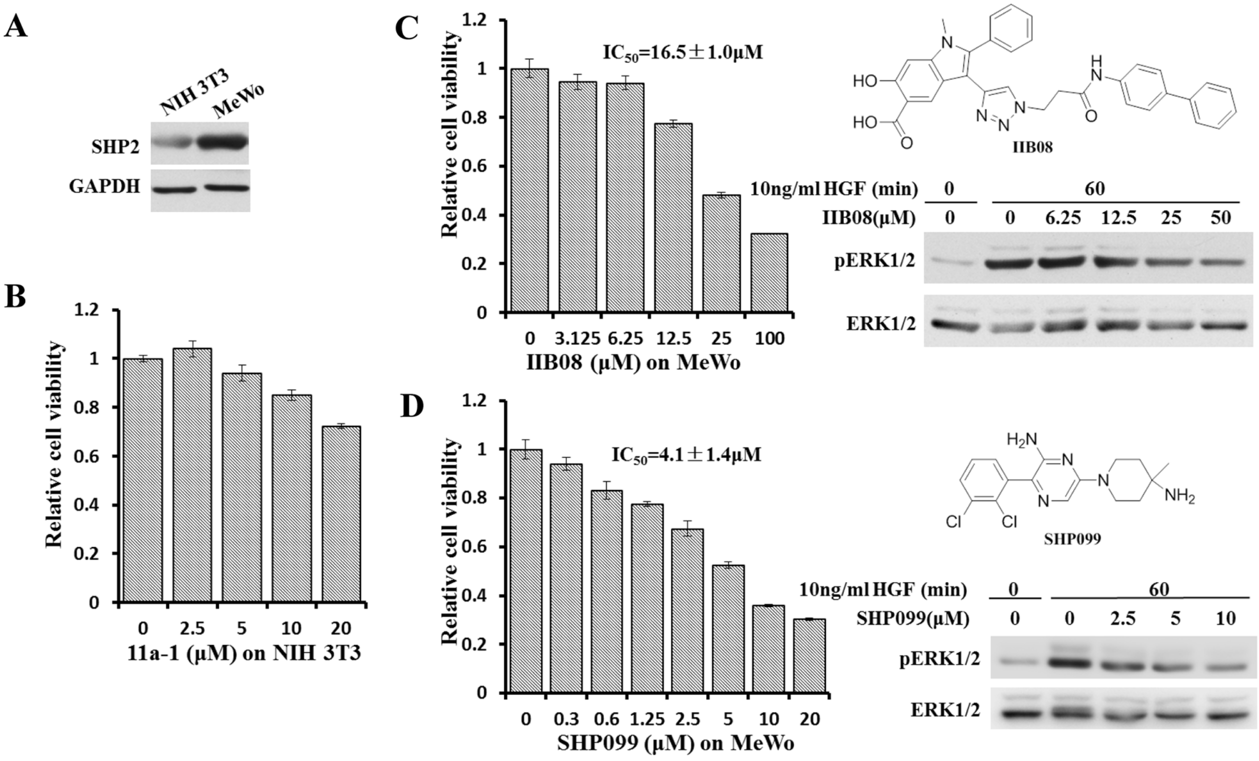

Supplementary Figure S2: 11a-1 inhibits MeWo cell viability through targeting SHP2.

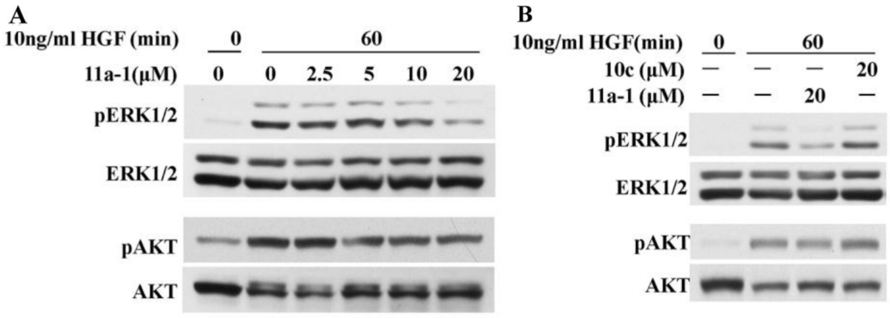

Supplementary Figure S3: A. SHP2 inhibitor 11a-1 inhibits HGF-induced REK1/2 and AKT activation in B16F10 mouse melanoma cell, but **B.** the negative control compound 10c doesn't show any inhibition on ERK1/2 or AKT activation in B16F10 cell line.

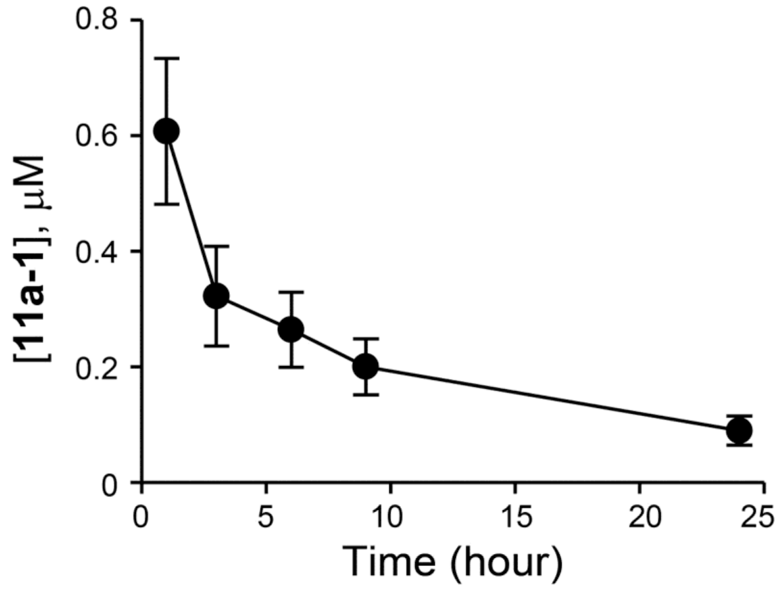

Supplementary Figure S4: Pharmacokinetics of 11a-1. Three mice were injected a single *IP* dose of 11a-1 at 10 mg/kg. At different time, blood samples were collected and analyzed by HPLC/mass spectrometry.

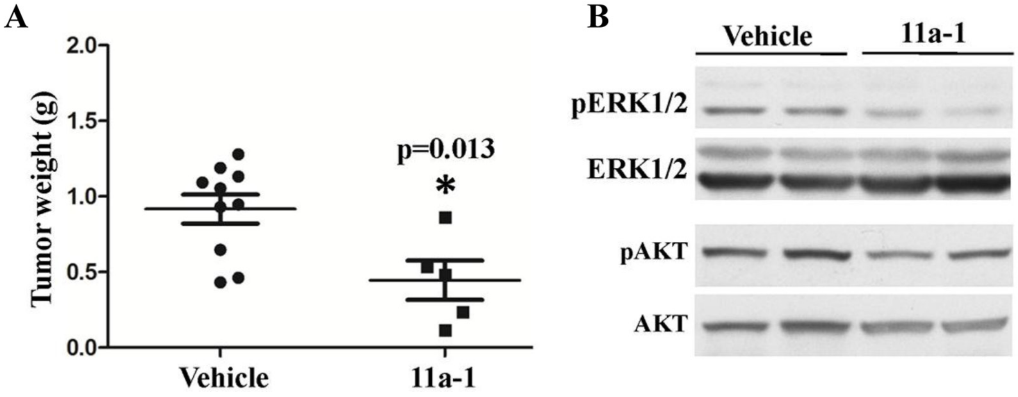

Supplementary Figure S5: 11a-1 suppresses B16F10 melanoma cell xenograft tumor growth *in vivo*. A. 11a-1 treatment significantly reduced tumor weight. **B.** 11a-1 treatment led to decreased ERK1/2 and AKT activation in B16F10 xenograft tumor samples.
